# Suppression of Belowground Volatiles in Maize Depends on Cover Crop Legacy and Genotype

**DOI:** 10.1007/s10886-025-01632-z

**Published:** 2025-08-27

**Authors:** Olivia W. Trase, Nathaniel McCartney, Jared G. Ali

**Affiliations:** 1 Department of Entomology, The Pennsylvania State University, University Park, PA 16802 USA; 2 Interdepartmental Degree Program in Ecology, The Pennsylvania State University, University Park, PA 16802 USA

**Keywords:** Volatile organic compounds, Belowground interactions, Root herbivory, Entomopathogenic nematodes, *Diabrotica virgifera virgifera*, *Steinernema feltiae*

## Abstract

Volatile organic compounds are important chemical signals involved in plant-insect interactions. In recent decades, volatiles have been used in many agricultural applications to help control crop pests, but fewer applications have been developed for belowground pests despite volatile signaling and olfactory cues being crucial for orientation and communication of belowground organisms. Volatile signals also depend heavily on soil characteristics which influence both production and diffusion of these volatile compounds. Cover cropping is one agricultural practice that significantly alters soil properties and has been shown to help control certain insect pests, but there is insufficient data on how cover cropping can affect belowground volatile production and recognition. In this study we explored constitutive and root herbivore induced total belowground volatile emissions in two commercial cultivars of maize (*Zea mays* L.) planted after different cover crop treatments as well as entomopathogenic nematode (EPN) foraging behavior toward plant cues. Contrary to expectation, we found that volatile emission was significantly reduced in response to herbivore feeding and this response was stronger when maize was planted after certain cover crops. In addition, infective juvenile EPN moved equally toward both herbivore-infested and control plants and were not affected by cover crop treatment. Our results suggest that plant responses to insect damage do not always result in adaptive indirect defenses: in some contexts, herbivores may actually reduce their visibility to natural enemies. Moreover, the plasticity and intensity of these responses depend on both plant genotype and soil legacy.

**Supplementary Information:**

The online version contains supplementary material available at 10.1007/s10886-025-01632-z.

## Introduction

Plants use chemical signaling for a variety of purposes, including intra-plant communication, repulsion of herbivores, and attraction of pollinators and natural enemies (Paré and Tumlinson 1999). Among the chemicals plants use for signaling are volatile organic compounds, or VOCs, which are secondary metabolites with low molecular weights and high vapor pressures at ambient temperatures (Paré and Tumlinson 1999). While thousands of VOCs have been characterized in the literature, much of the focus has been on how these volatiles facilitate aboveground interactions. Understanding volatile-mediated interactions is arguably more important belowground, where visual signals are virtually nonexistent and olfactory cues are often the primary forms of communication and orientation for soil organisms (Johnson and Nielsen 2012).

Both plants and soil organisms produce volatiles belowground. In plants, root volatiles can indicate the suitability of the root for herbivore feeding, communicate with other parts of the plant during pest or pathogen attacks, and signal the presence of herbivores to natural enemies in the soil (Wenke et al. 2010). Passively produced volatiles are considered to be ‘constitutively produced’ VOCs, while volatiles emitted as a result of pathogen or pest attack are termed ‘induced’ VOCs. Both constitutive and induced root volatiles play important ecological roles belowground, from shaping the structure of the rhizosphere microbiome to alerting nearby plants and soil-dwelling arthropods and nematodes to the presence of a specific root herbivore (Wenke et al. 2010; Rowen and Kaplan 2016; Davidson-Lowe et al. 2024).

Entomopathogenic nematodes are an extremely promising area of research for control of root-feeding pests due to their ability to kill most insect orders and the ease of culturing them (Ali and Davidson-Lowe 2015). These organisms locate their prey using chemosensation and chemotaxis, whereby they enter their insect hosts through an external orifice or through penetration of the cuticle (Lewis et al. 2006; Rasmann et al. 2012). EPNs have a mutualistic relationship with an entomopathogenic bacterium that resides in their intestine which they release into their insect host upon entry (Chaston et al. 2011). The bacteria kill the host and proliferate inside the cadaver, providing food for the EPN which are bacteriophagic in nature (Ali and Davidson-Lowe 2015). The EPN are then able to reproduce inside the cadaver before exiting the host as infective juveniles, the only life stage of the nematode that can survive outside hosts for extended periods of time (Gaugler 2002). Finally, they search for new hosts using chemotactic cues.

EPN are often categorized into two main groups based on prey-finding strategies: cruising foragers, which are very mobile, and ambushers, which employ a sit-and-wait strategy. Cruisers tend to have higher success in finding sedentary and hidden resources while ambushers have more success with mobile prey (Lewis et al. 2006). However, recent research suggests that most EPN species’ foraging strategies exist along a continuum between ambushing and cruising and could be more plastic depending on the situation (Campbell and Gaugler 1997; Lewis et al. 2006; Ali and Davidson-Lowe 2015).

While laboratory experiments have been able to reveal much about the life history and foraging strategies of these organisms, understanding their chemotactic cues is still a very active area of research. Early work indicated that carbon dioxide was the main signal for prey finding and aggregation, perhaps in combination with some more specific cues, but scientists later argued that the EPNs could use different cues for short-range vs. long-range chemotaxis (Ali and Davidson-Lowe 2015). These herbivore-induced plant volatiles (HIPVs) are increasingly recognized as key cues for EPN host-finding and form part of a plant’s indirect defense strategy. In 2005, Rasmann et al. demonstrated in the lab and field that European maize varieties release (*E*)-β-caryophyllene after feeding by western corn rootworm larvae which in turn attracts the EPN *Heterorhabditis megidis*, a cruising forager (Rasmann et al. 2005; Hiltpold et al. 2011). These experiments offer a first look at how we might be able to optimize plant and soil properties for improved control of belowground pests using entomopathogenic nematodes.

Production and diffusion of volatile cues belowground can be mediated by a variety of factors including nutrient and oxygen availability, soil moisture, temperature, pH, and soil texture (Insam and Seewald 2010). This is particularly relevant in agricultural systems, where much of the agricultural practices in use today are constantly altering soil properties thereby affecting the way that volatiles facilitate belowground interactions. Despite the demonstrated importance of belowground volatiles in agriculturally relevant ecological interactions, like pest resistance and interspecific competition, little research has focused on how changes to soil due to agricultural practices affect volatile production.

Cover cropping, or planting non-commodity crops in between growing seasons, has regained popularity in recent years for farmers to improve soil health. Cover cropping has long been used to improve soil conditions (Lal 2015), including preventing soil erosion, increasing water retention, reducing leaching, and suppressing weeds (Langdale et al. 1991; Meisinger et al. 1991; Morse 1993; Teasdale 1996). More recently, certain cover crops have been shown to influence plant defense responses, including resistance to herbivores and pathogens (Lundgren and Fergen 2010; Rivers et al. 2018), raising the possibility that they May also affect signaling via volatile organic compounds. In fact, Davidson et al. 2021 found that maize planted in pea- and radish-conditioned soil had significantly higher quantities of aboveground volatiles compared to maize planted in triticale-conditioned soil or in fallow soil (Davidson-Lowe et al. 2021).

Of the three main categories of cover crops – legumes, grasses, and non-legume broadleaves – each are used for specific agronomic benefits (Koudahe et al. 2022). Legumes contain nitrogen-fixing bacteria that provide fixed nitrogen back into soil for subsequent crops and field pea has often been used for this purpose (Murrell et al. 2017; Davidson-Lowe et al. 2021). Dual-purpose cover cropping, or using the same crop for soil management and forage production, has gained traction in recent years and triticale, a grass, has emerged as a strong candidate crop (Glaze-Corcoran et al. 2023). Radish, a non-legume broadleaf, has been used for weed suppression as well as for its ability to root deeply and create access to further N reservoirs (Murrell et al. 2017). Because cover crops alter nutrient availability, microbial communities, and root exudates, they may also affect the biosynthesis or release of root volatiles. However, their specific influence on constitutive and herbivore-induced volatile profiles in subsequent crops remains poorly understood.

To address this knowledge gap, we examined how legacy effects from five different cover cropping treatments influence both constitutive and herbivore-induced volatile emissions in maize roots. As volatile profiles can also vary by maize genotype (Degen et al. 2004), we tested two commercial cultivars, using western corn rootworm (WCR; *Diabrotica virgifera virgifera*) as a model specialist herbivore. We hypothesized that cover crop species would affect both constitutive and WCR-induced belowground volatile production, but that volatile emissions would increase in WCR-infested treatments regardless of cover crop legacy. We also expected entomopathogenic nematodes to recognize and orient toward WCR-induced volatile signals.

## Materials and methods

*Experimental Design and Growing Conditions.* In the first experiment, our aim was to assess differences in belowground volatile profiles of maize in soil conditioned with various cover crop treatments and infested with western corn rootworm. In the spring of 2021, we collected soil from our cover crop research site (40.723 N 77.928 W) at the Russell E. Larson Agricultural Research Center (Pennsylvania Furnace, PA, USA). The cover crop species used represented three functional cover crop groups: legume, non-legume broadleaf, and grass. Soil was collected after the cover crops had been tilled from four field plots each (from a randomized complete block design) that had been planted with a legume (pea; *Pisum sativum*), a non-legume broad-leaf (radish; *Raphanus sativus*), a grass (triticale; x*Triticosecale*), or a mixture containing each functional group designed for improved nitrogen management and biomass production (pea; triticale; red clover, *Trifolium pratense*; canola, *Brassica napus*) (Murrell et al. 2017), as well as four plots that had been left fallow. For each treatment, the soil from the four plots was mixed and stored at 4 °C.

The experiment was conducted from July 2021 to October 2021 in five blocks staggered by two-week increments. We used two organic maize hybrids (Master’s Choice 90-day MC4050 and Blue River Organic 98-day 42C87) to examine potential differences in cultivars due to the high genetic and phenotypic diversity of maize (Kuleshov 1933). Maize seeds were surface sterilized by immersion in a 15% household bleach solution containing 0.01% Triton X-100 for ten minutes followed by three washes with DI water to reduce variation due to surface contamination. Seeds were germinated on paper towels for four days at room temperature, germinated seeds were then planted in soil from each of the five cover crop treatments in 10 cm by 12 cm square pots. Maize plants were grown for five weeks in greenhouse conditions (16 h:8 h L: D, 22–25 °C, 63–67% RH) and watered every other day. Once maize plants had reached fifth vegetative stage, twenty neonate WCR larvae were added to half of the plants in each cover crop treatment (*n* = 20). 48 h after infestation, belowground volatile profiles were collected from both WCR-treated and control pots.

In the second experiment we compared attraction of the entomopathogenic nematode *Steinernema feltiae* toward WCR infested roots of maize planted after pea, radish, triticale, or fallow in a series of choice assays. In the springs of 2022 and 2023, soil was collected and stored in the same way as the previous experiment and MC4050 seed sterilization, germination, and greenhouse conditions also followed the previous experimental protocol. Maize plants were grown for two weeks and watered every other day prior to beginning the choice assay.

*Volatile Collection.* Perforated aluminum tubes (0.253 in. ID) were carefully inserted into the soil and attached to VOC traps containing 30 mg adsorbent HaySep Q. Using a vacuum pump, air was pulled from soil and through the VOC trap at a rate of approximately 100 ml per minute for 24 h. Volatiles were then eluted from each VOC trap using 150 µl dichloromethane, and 5 µl of an internal standard containing 40ng/µl nonyl acetate and 40ng/µl n-octane was added to each sample. Vials with eluted samples were stored at −20 C. Samples were then injected and analyzed by gas chromatography mass spectrometry (GC/MS) using an Agilent 7890 gas chromatograph coupled to a 5977 mass spectrometer operated in positive EI mode using typical tune parameters. Injections of 1 µl were made in splitless mode with the inlet held at 230 °C and using a helium carrier gas flow of 0.8 mL/min. The column (HP-5MS, 30 m, 0.25 mm id, 0.25 μm film thickness; Agilent, USA) was maintained at 40 °C for 2 min, increased by 10 °C/min increments to 300 °C, and then held for 4 min. Peaks were isolated by deconvolution using MassHunter Unknowns Analysis (Agilent Technologies, USA) and target compounds were identified using mass spectral data from the NIST 17 and Adams spectral libraries (Adams 2007), published retention indices (“The Pherobase: Database of pheromones and semiochemicals”), and, when possible, authentic standards. Total VOC content, individual compounds, and volatile profiles were compared.

**Fig. 1:**
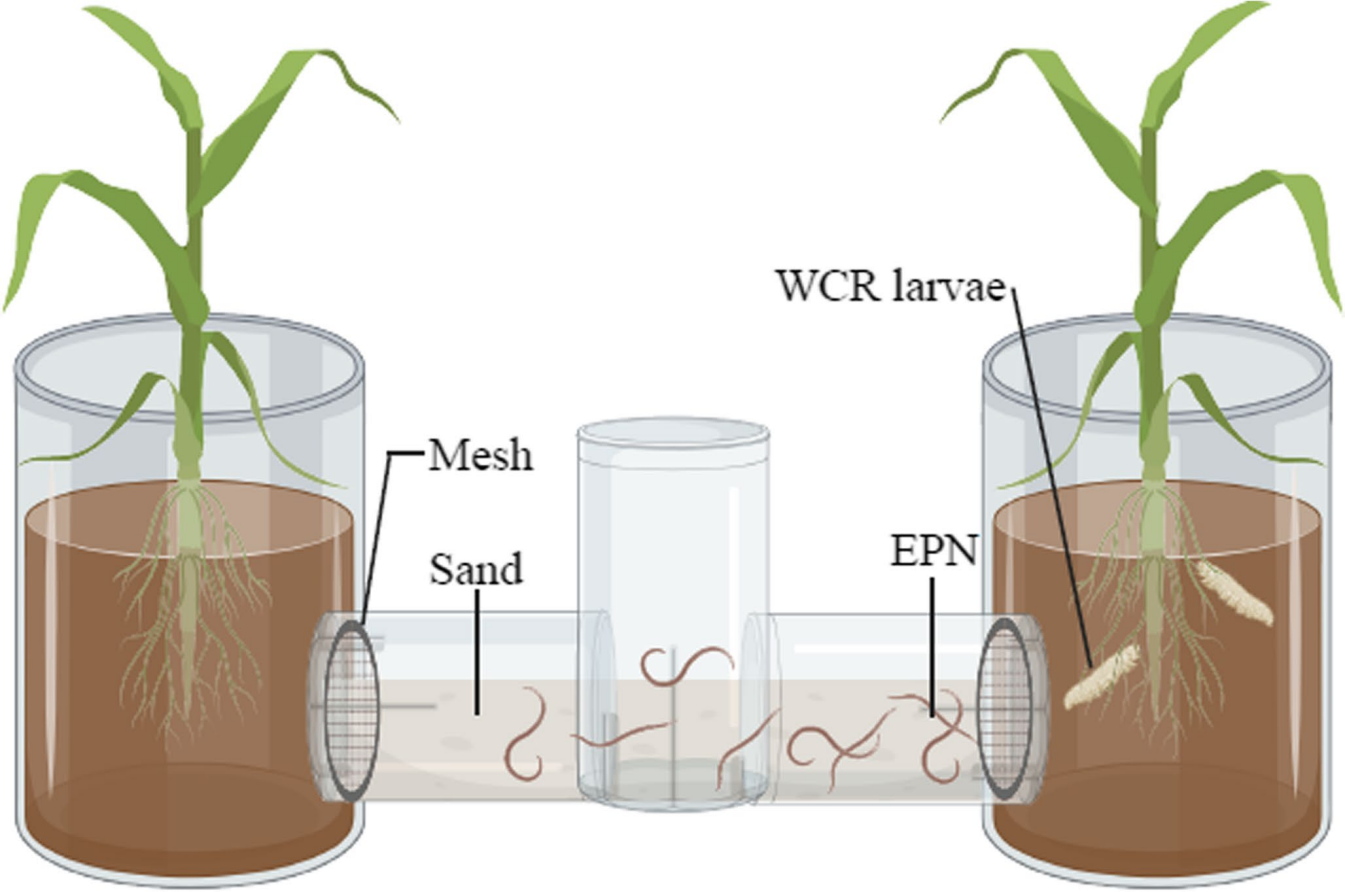
Diagram depicting the two-choice olfactometer

*Entomopathogenic Nematode Choice Assays.* For the choice assay, a pair of maize plants from the same cover crop treatment were transplanted into two glass pots (*d* = 5 cm, *h* = 12 cm). After an initial acclimation period overnight, one plant in each pair was infested with ten neonate WCR larvae. After six hours of feeding, the pots in each pair were joined using a cylindrical glass connector (*d* = 2.5 cm, *l* = 6 cm) filled with sterile pool filter sand and fitted with Teflon bushings containing a fine mesh filter (25-micron stainless steel, TWP, Inc., Berkeley, CA, USA). This system allowed the nematodes access to either side of the cylindrical glass connector but prevented them from encountering plant roots or WCR (Fig. 1). Approximately 500 naïve *S. feltiae* infective juveniles were added to the central opening of the connector and given 48 h to move to either side of the olfactometer. *S. feltiae* are known to attack WCR larvae and utilize a foraging strategy that is intermediate between ‘ambusher’ and ‘cruiser’ strategies (Gaugler 2002), and were sourced from our lab-maintained colony on *Galleria mellonella* host. Sand from each side of the olfactometer was deposited in separate Baermann funnels, where active EPN had 72 h to move into the water trap. EPN were then counted under a dissecting microscope. This assay was repeated for each cover crop treatment in both 2022 (*n* = 9) and 2023 (n = F:8, P:7, R:6, T:7) and completed in two experimental blocks per year.

We chose not to conduct EPN choice assays across cover crop soils, as such comparisons are unlikely to occur in real field conditions where soil legacies are typically homogenized. Instead, we focused on how cover crop-conditioned soils affect the apparency of root-feeding larvae to natural enemies, which better reflects ecologically relevant scenarios.

Approximately 500 entomopathogenic nematodes (*S. feltiae*) were introduced into the central opening and given 48 h to move to one of the two attached glass containment chambers which held either a control maize plant or a maize plant infested with ten neonate western corn rootworm.

*Statistical Analysis.* All statistical analyses were performed in the R statistical environment using RStudio (R v. 4.2.1 RStudio Team 2020) or Python (Python Software Foundation. Python Language Reference, version 3.8.3. Available at http://www.python.org). Details on the specific packages used in the analysis can be found in Appendix S2.

Chromatogram peak areas for each sample were converted to nanograms using the TIC peak area of the internal standard nonyl acetate. Contaminants were considered to be any siloxanes, xylene, toluene, halogenated compounds, phthalates, homosalate, or other common synthetically-produced compounds and were removed from the analysis.

Total VOC content was calculated as the sum of each peak detected in each chromatogram and each total was normalized by the internal standard, nonyl acetate, and log-transformed to meet normality assumptions. Differences in total VOC content based on maize variety, cover crop treatment, and WCR infestation status were assessed using a linear mixed effects model followed by an ANOVA where variety, cover crop treatment, and WCR status were fixed effects, and experimental block was a random effect. Differences in total VOC content between infested and non-infested plants of each treatment were assessed using a Student’s T-test. Differences in volatile profiles were assessed using PERMANOVA and PERMDISP.

For analysis of individual compounds, outliers were considered to be either compounds that appeared in fewer than three samples or samples that contained fewer than three compounds total. In addition, compounds that were not found in any samples during any one of the experimental blocks were considered to be environmental and were also removed from subsequent analysis. For both individual compounds and chemical classes, differences between variety, cover crop treatment, and WCR infestation status were assessed using a generalized linear mixed model using a negative binomial distribution followed by a type II ANOVA. Generalized linear mixed models using a negative binomial distribution were also used to assess differences between WCR-infested and control plants in the same variety and cover crop legacy.

Prediction of WCR-feeding based on volatile composition was assessed using a random forest classification algorithm. Data was split into training data (80%) and testing data (20%). Data were normalized within each block by multiplying each value by some constant such that the average total nanograms of volatiles for each block was the same as the average total nanograms of volatiles for the entire experiment. To improve the model, only volatiles that showed a correlation greater than 25% with WCR presence were included. Given the high levels of multicollinearity among volatiles, the threshold for variance inflation factors was set to 10. The performance of random forest models was evaluated using the recall score, defined as the ratio of correctly identified WCR-infested observations to the total number of actual WCR-infested observations, and accuracy, which is the proportion of correctly identified observations out of the total number of observations. Best parameters for the random forest algorithm were selected using a grid search and ten iterations of cross-validation.

For the second experiment, differences between total number of EPN recovered were assessed using ANOVA. Sample pairs for which fewer than 10 EPN were recovered in total were removed from the analysis.

Chemotaxis indices were calculated by subtracting EPN that moved toward control plants from EPN that moved toward WCR-infested plants, divided by total EPN recovered. Differences in average chemotaxis index between cover crop treatments were assessed using ANOVA. Fixed effects included cover crop treatment, year, and the difference in biomass between the two plants while random effects included the olfactometer orientation and the experimental block.

- Differences in the number of EPN moving toward infested versus control plants for each cover crop treatment were assessed using a generalized linear mixed effects model with a Poisson distribution. Fixed effects included WCR infestation status and plant biomass, and random effects included the orientation of the olfactometer, the pair, and the experimental block.

## RESULTS

**Fig. 2:**
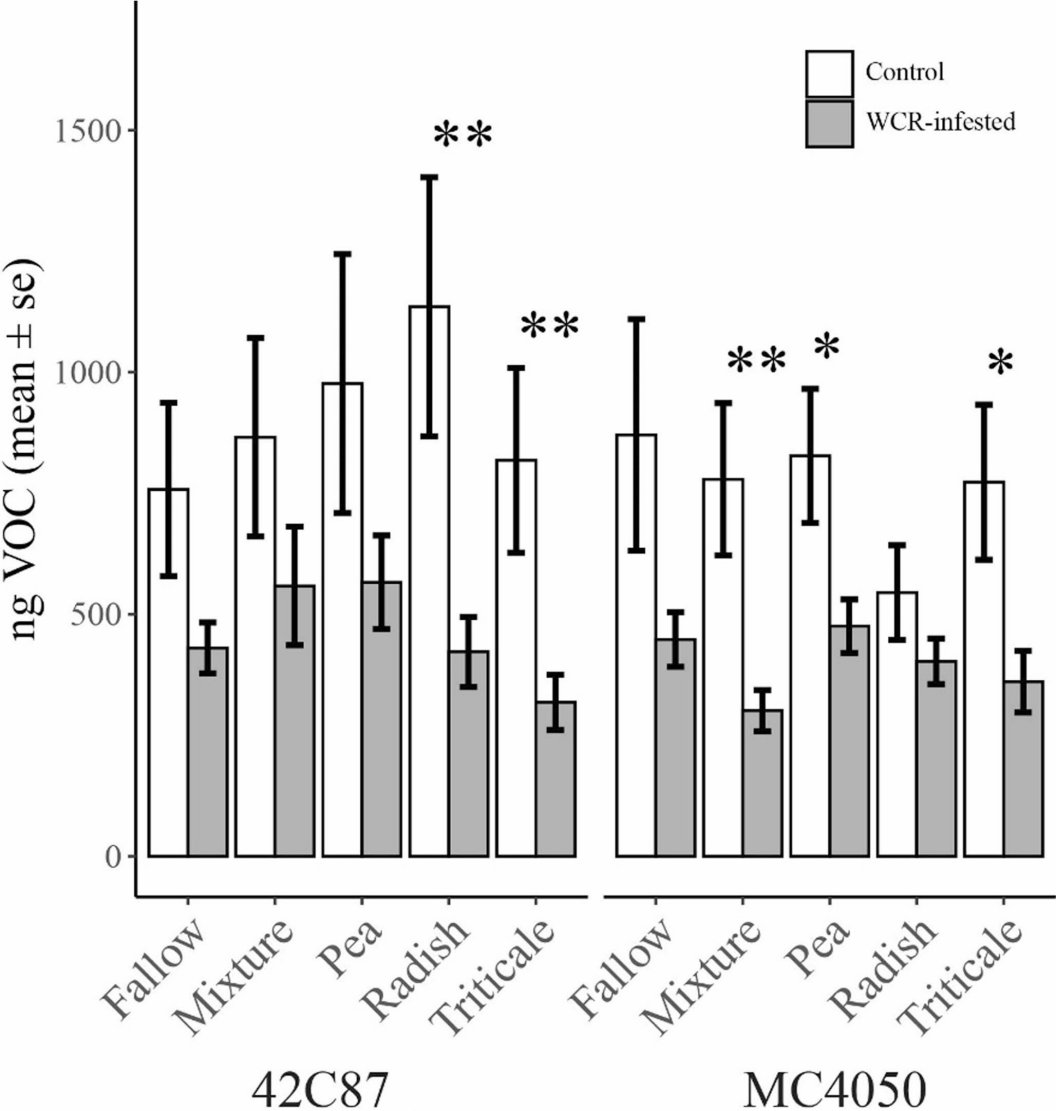
Average total belowground volatile content for each maize variety and cover crop soil treatment

*Total Volatile Content.* The results showed that total volatile content was significantly lower in WCR-infested treatments compared to control treatments (F_(1,376)_ = 31.85, *p* < 0.001). Figure 2 shows pairwise differences between WCR-infested and control treatments for each variety and each cover crop treatment. There was no significant difference between total WCR-infested and control belowground volatile production in the fallow treatment in either variety (Fig. 2, MC4050: F_(1,33)_ = 3.06, *p* = 0.09; 42C87: F_(1,33)_ = 0.032, *p* = 0.86). Pairwise post-hoc tests indicated that control volatile production was significantly higher than WCR-infested volatile production for both varieties planted in triticale-conditioned soil (MC4050: F_(1,32)_ = 6.97, *p* = 0.01; 42C87: F_(1,33)_ = 8.16, *p* = 0.007), for 42C87 planted in radish-conditioned soil (F_(1,33)_ = 12.7, *p* = 0.001), for MC4050 in pea-conditioned soil (F_(1,33)_ = 5.49, *p* = 0.03) and for MC4050 planted after the cover crop mixture (F_(1,33)_ = 11.87, *p* = 0.002). Within WCR-infested treatments, there was no significant difference between cover crops (MC4050: F_(4, 90)_ = 1.79, *p* = 0.14; 42C87: F_(4, 91)_ = 1.58, *p* = 0.19). The same was true for control treatments (MC4050: F_(4, 86)_ = 1.64, *p* = 0.17; 42C87: F_(4, 84)_ = 1.02, *p* = 0.4).

Volatile content was normalized by the internal standard (nonyl acetate) and is here expressed in terms of nanograms of compound. Bars represent ± 1 SEM. Stars indicate where the difference between control (white) and WCR infested treatments (gray) was significant (*p* < 0.05*, *p* < 0.01**).

*Volatile Composition.* Volatile composition was significantly different between WCR-infested and control treatments (pseudo-F_(1,367)_ = 9.74, R^2^ = 2.5%, *p* < 0.001). Differences in volatile composition between cover crop treatments or variety were not significant (Cover Crop: pseudo-F_(4,367)_ = 0.86, R^2^ = 0.9%, *p* = 0.15; Variety: pseudo-F_(1,367)_ = 0.78, R^2^ = 0.2%, *p* = 0.29). Dispersion was not different between WCR treatments or varieties (WCR: F_(1,385)_ = 0.63, *p* = 0.39; Variety: F_(1,385)_ = 0.8, *p* = 0.31), and it was marginally different between cover crop treatments (F_(4,382)_ = 1.61, *p* = 0.07).

**Fig. 3:**
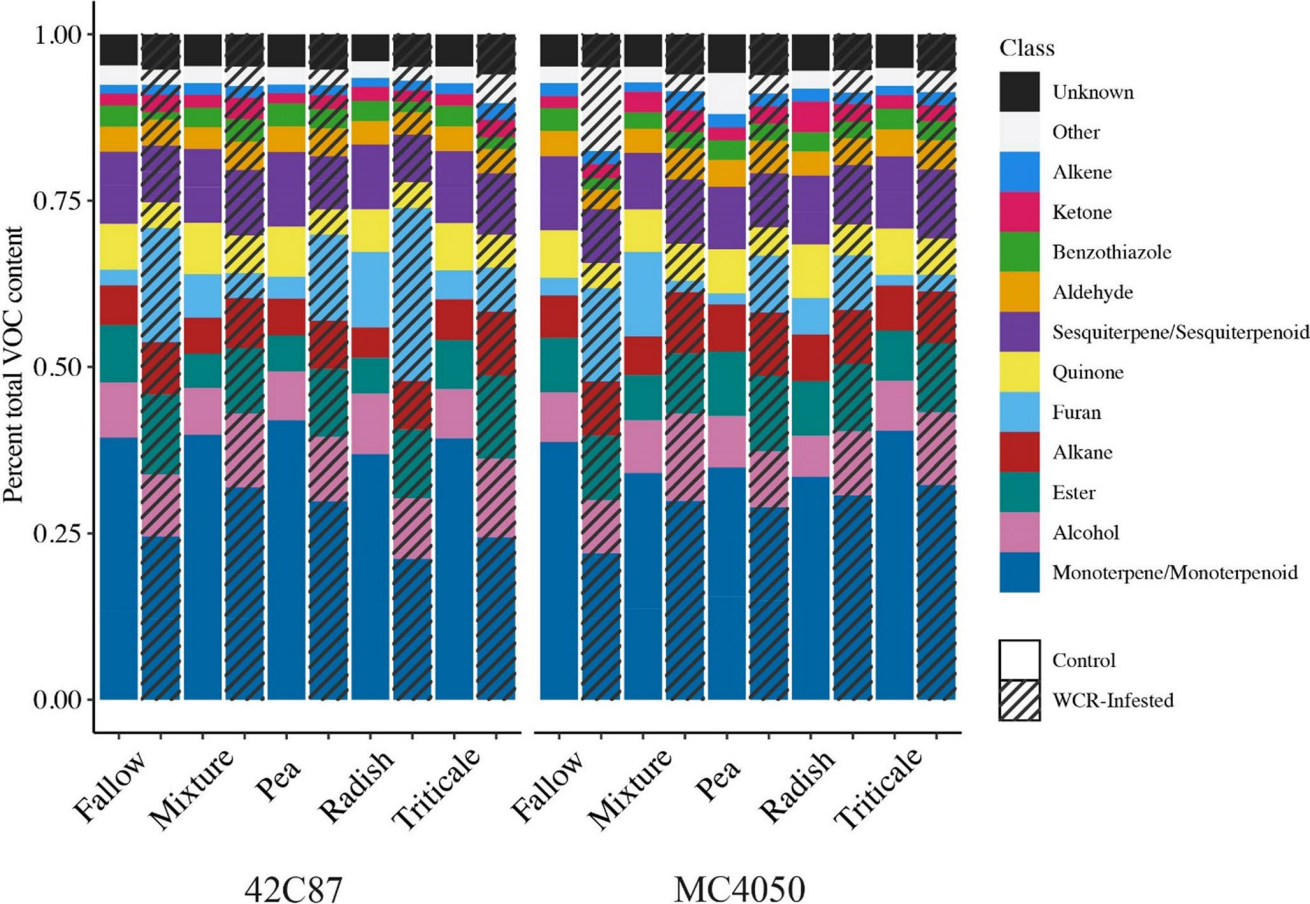
Percent total volatile content of each chemical class for each maize variety and cover crop soil treatment

*Volatile Analysis by Chemical Classification.* Identified volatiles were classified into alcohols, aldehydes, alkanes, alkenes, benzothiazoles, esters, furans, ketones, monoterpenes/monoterpenoids, quinones, salicylates, sesquiterpenes/sesquiterpenoids, unknowns, and other (Fig. 3).

WCR-infested treatments are denoted by diagonal black lines.

All chemical classes except furan (*X*^2^_(1, *N* =375)_ = 0.0009, *p* = 0.98) were detected in significantly lower quantities in WCR-infested treatments compared with controls (*p* < 0.001). Alcohols differed significantly between maize varieties (*X*^2^_(1, *N* =375)_ = 7.98, *p* = 0.005) as did furans (*X*^2^_(1, *N* =375)_ = 6.1, *p* = 0.013) and sesquiterpenes (*X*^2^_(1, *N* =375)_ = 3.88, *p* = 0.049). In plants that were WCR-infested, aldehydes, benzothiazoles, furans, and monoterpenes were significantly different between cover crop treatments (aldehyde: *X*^2^_(4, *N* =188)_ = 9.62, *p* = 0.047; benzothiazole: *X*^2^_(4, *N* =188)_ = 11.9, *p* = 0.02; furan: *X*^2^_(4, *N* =188)_ = 13.88, *p* = 0.008; monoterpene: *X*^2^_(4, *N* =188)_ = 9.78, *p* = 0.044). In control plants, furans and ketones were significantly different between cover crop treatments (furan: *X*^2^_(4, *N* =177)_ = 22.7, *p* = 0.0001; ketone: *X*^2^_(4, *N* =177)_ = 15.38, *p* = 0.004).

Linear mixed effects models were used to determine whether concentrations of each chemical class were significantly different between control and WCR-infested treatments. Boxes in blue indicate a significant reduction in concentration after WCR-infestation while red boxes indicate an increase in concentration after WCR-infestation. Darker colors denote lower p-values. Stars indicate significance after Benjamini-Hochberg adjustment for false discovery rate (*p* < 0.05*, *p* < 0.01**, *p* < 0.01***).

**Fig. 4:**
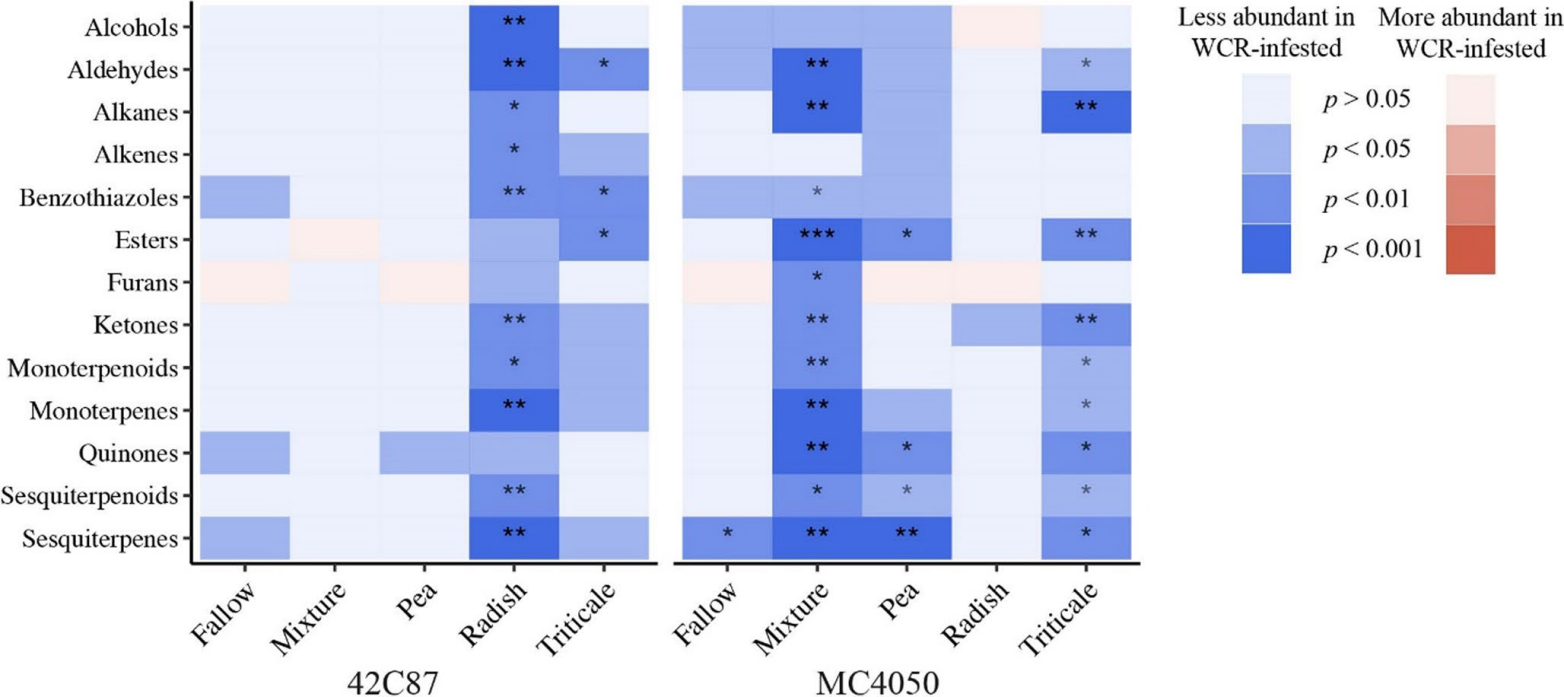
Heatmap displaying significant chemical classes

In most volatile chemical classes, 42C87 maize planted in radish conditioned soil and MC4050 maize planted in the cover crop mixture and triticale conditioned soils had significantly reduced volatile content after WCR infestation (Fig. 4).

**Fig. 5:**
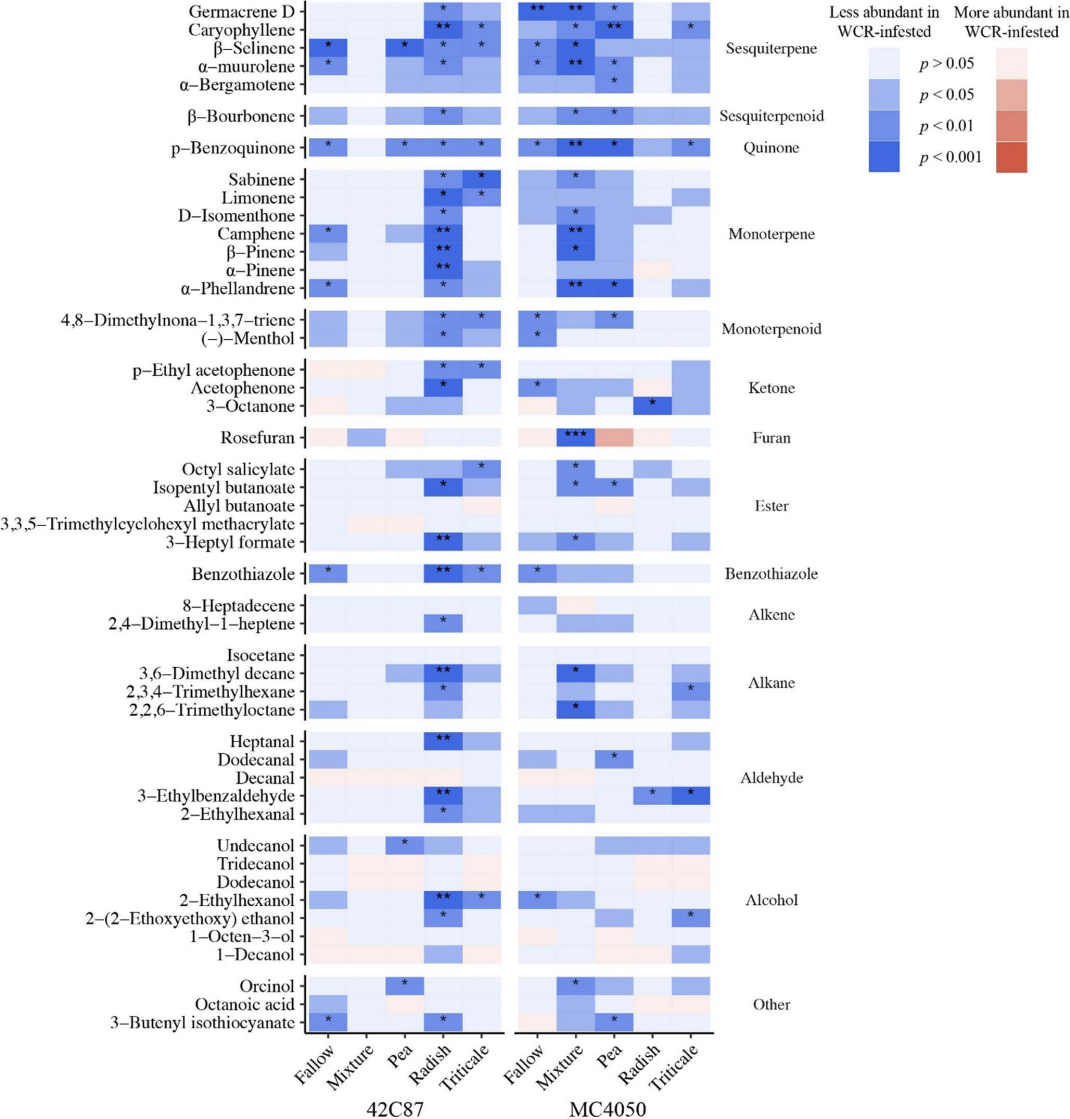
Heatmap displaying significant volatile compounds

*Volatile Analysis by Individual Compounds.* When comparing WCR-infested volatile content to control volatile content for individual compounds, WCR-infested volatiles were significantly lower than control levels only after certain treatments. For most compounds, the WCR-infested volatile content in 42C87 was significantly lower than the constitutive levels following the radish cover crop, whereas no such difference was observed in MC4050 (Fig. 5, *p* < 0.05). In contrast, WCR-infested volatile content was lower than constitutive levels for a majority of compounds in cover crop mixture conditioned soil in MC4050 but not for 42C87 (Fig. 5, *p* < 0.05). Notably, there were very few compounds that showed any difference in WCR-infested vs. control volatile production after the fallow or pea cover crops in either variety.

Linear mixed effects models were used to determine whether concentrations of each compound were significantly different between control and WCR-infested treatments. Boxes in blue indicate a significant reduction in concentration after WCR-infestation while red boxes indicate an increase in concentration after WCR-infestation. Darker colors denote lower p-values. Stars indicate significance after Benjamini-Hochberg adjustment for false discovery rate (*p* < 0.05*, *p* < 0.01**, *p* < 0.01***).

**Fig. 6:**
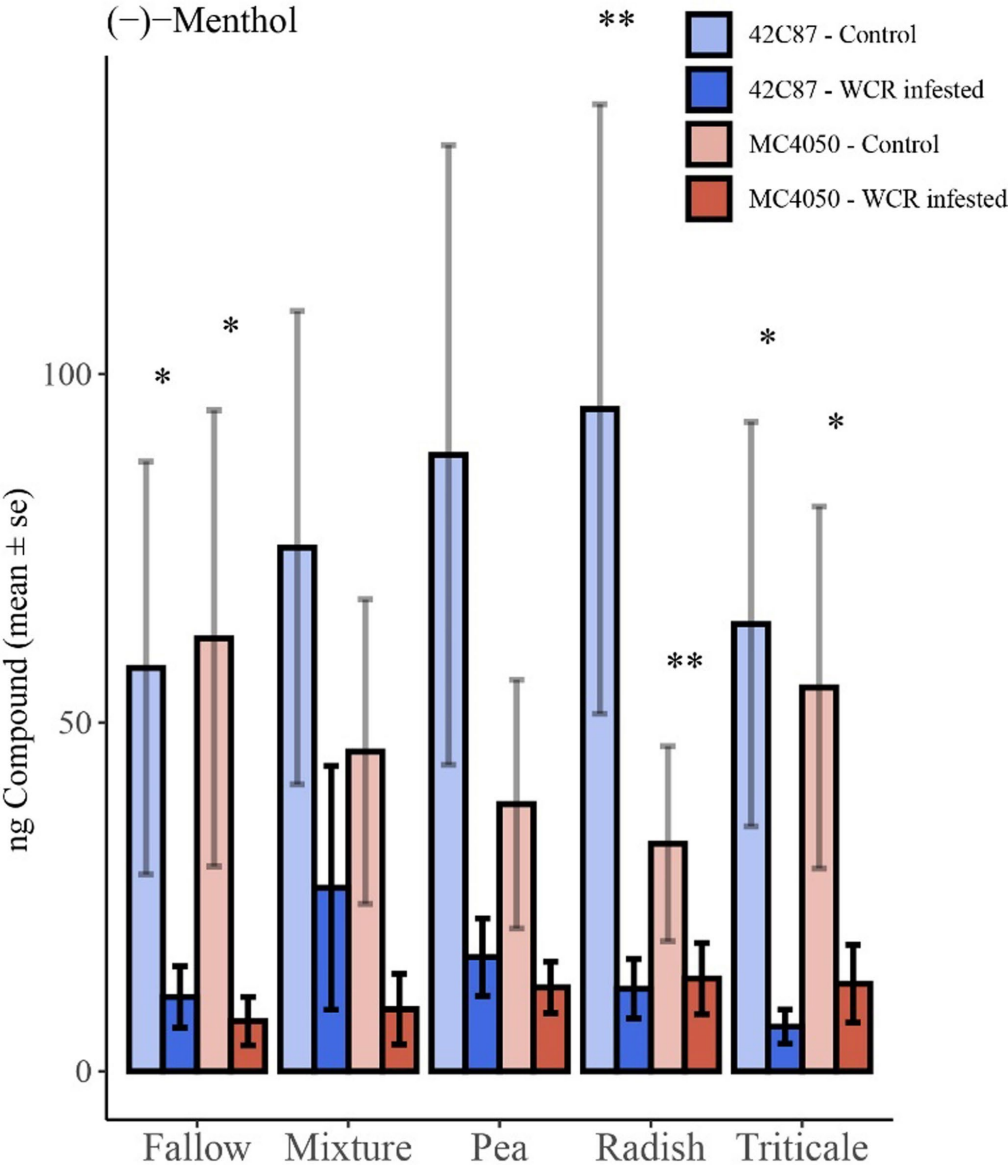
Bar plot displaying average quantities of Menthol in each treatment

*Random Forest Classification.* To understand whether certain compounds were strong predictors of WCR-infestation, compounds that had a high correlation with WCR-infestation status were used to train a random forest classification model. The model was able to predict the presence of WCR larval feeding with 98% recall and 64% accuracy using only one of the identified compounds: menthol (Fig. 6). Menthol was found in significantly lower levels in WCR-infested treatments compared to control treatments (X^2^_(1, *N* =375)_ = 19.58, *p* < 0.001).

Volatile content was normalized by the internal standard (nonyl acetate) and is here expressed in terms of nanograms of compound. Bars represent ± 1 SEM. Stars indicate where the difference between control (white) and WCR infested treatments (gray) was significant (*p* < 0.05*, *p* < 0.01**, *p* < 0.001 ***).

*Total EPN Recovery.* There was no difference between cover crop treatments in terms of the total number of *S. feltiae* recovered from the traps (X^2^_(3, *N* =52)_ = 1.38, *p* = 0.71). On average, 143 ± 10 out of the 500 nematodes moved toward either side of the olfactometer and were recovered in the Baermann funnels.

**Fig. 7:**
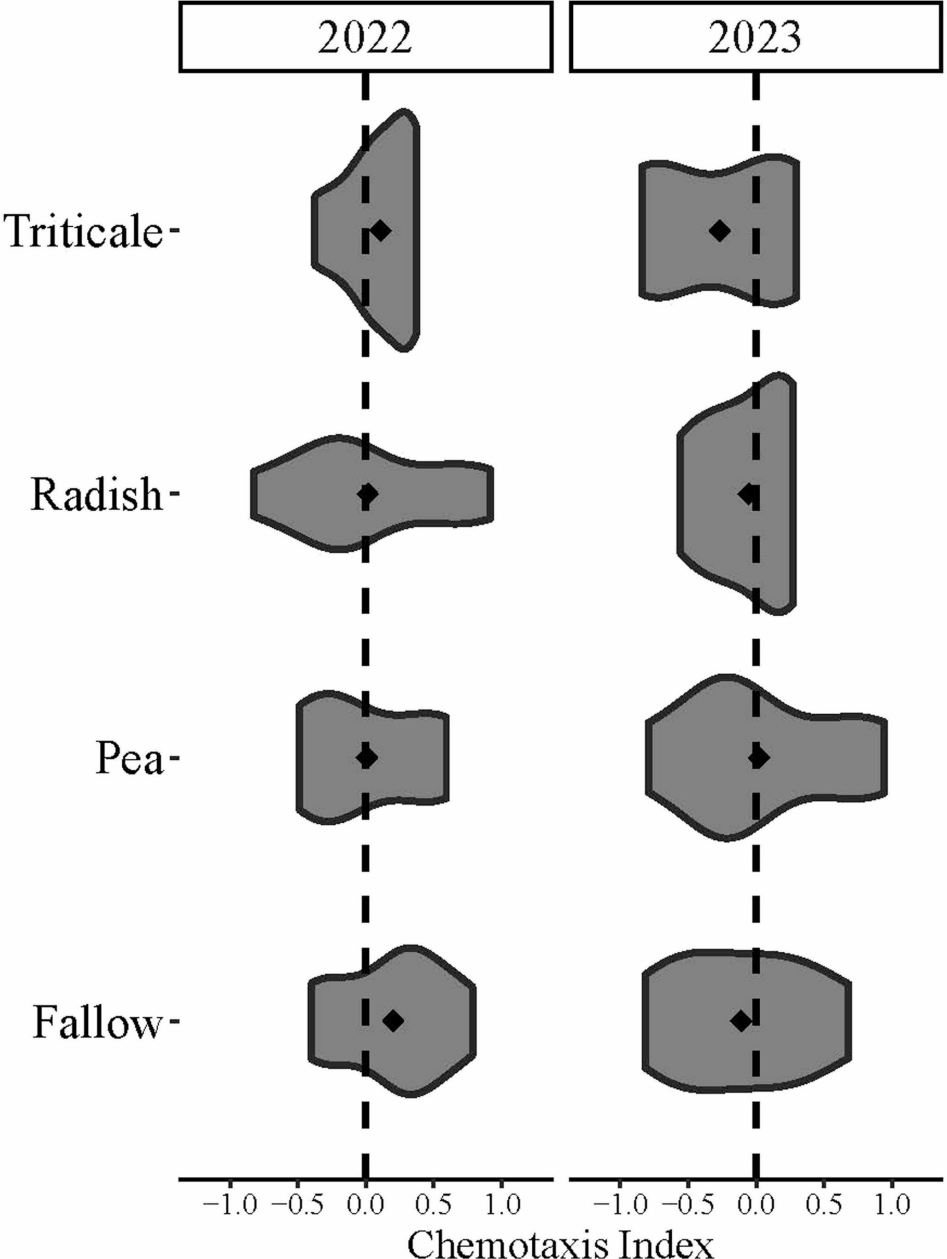
Violin plot of the chemotaxis indices for *S. feltiae* under different cover crop treatments and years

*Chemotaxis Indices.* Chemotaxis indices for *S. feltiae* were not different based on cover crop treatment (X^2^_(3, *N* =52)_ = 0.46, *p* = 0.93) nor were they different from year to year (Fig. 7, X^2^_(1, *N* =52)_ = 0.72, *p* = 0.4).

There were no significant differences in *S. feltiae* chemotaxis indices based on cover crop treatment or year. Chemotaxis indices were calculated by subtracting the number of nematodes that moved toward the control plant from the number of nematodes that moved toward the WCR-infested plant, all divided by the total number of nematodes recovered. The dotted line indicates a chemotaxis index of zero, indicating that there were equal numbers of *S. feltiae* going toward each plant in the assay. Points indicate the mean chemotaxis index.

**Fig. 8:**
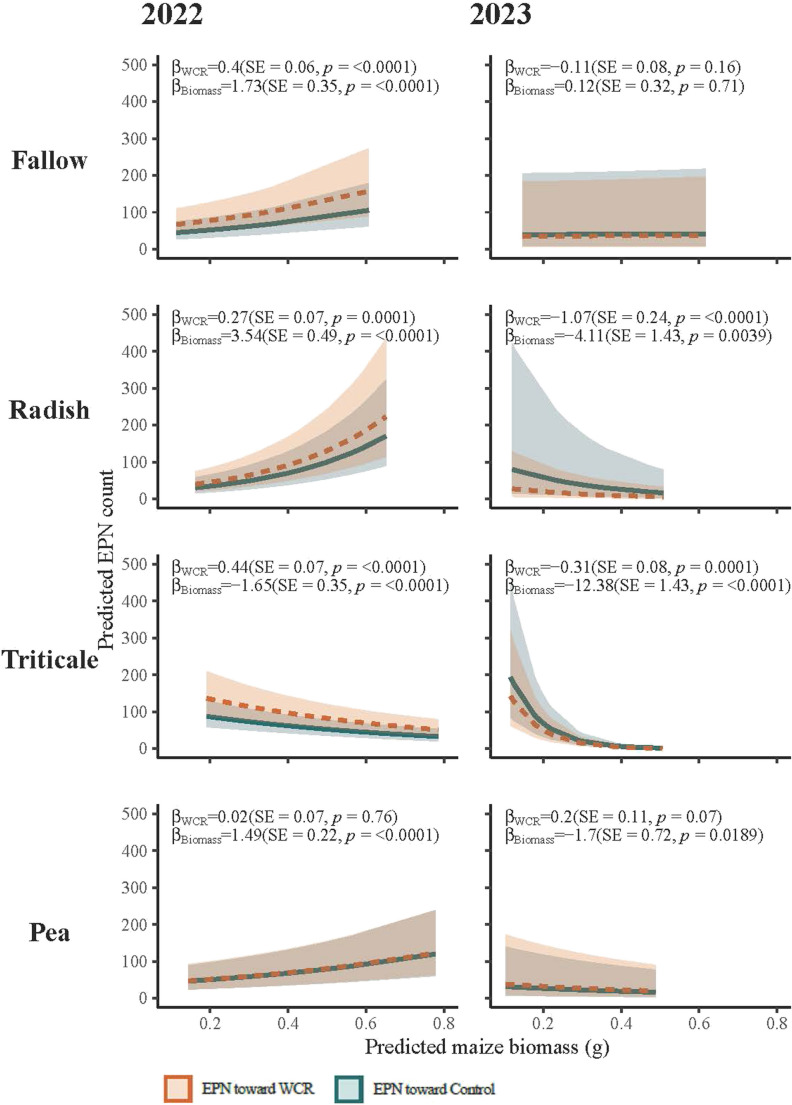
Predicted counts of *S. feltiae* as a function of maize biomass and WCR infestation status according to generalized linear mixed effects models

*EPN Movement Toward WCR-Infested Plants.* Generalized linear mixed effects models run for each cover crop treatment and each year showed that both WCR infestation and plant biomass were predictors for EPN movement (Fig. 8). In 2022, WCR infestation was a significant positive predictor of EPN movement in the fallow, radish, and triticale treatments, whereas in 2023 it was a negative predictor in the triticale and radish treatments. Greater plant biomass was a significant positive predictor in the fallow, radish, and pea treatments in 2022, but was a significant negative predictor for triticale. In 2023, plant biomass was a negative predictor for EPN movement in radish, triticale, and pea. Overall, *S. feltiae* were no more likely to move toward WCR-infested plants compared to control plants, or toward larger plants compared to smaller plants, but these results depended on the cover crop treatment and the year the soil was collected.

Predictions of *S. feltiae* movement were not consistent across cover crop treatment or soil collection year. Models used Poisson distributions where plant biomass and WCR infestation are fixed effects and olfactometer orientation, plant pair, and experimental block are random effects. Statistical models show predictor values for WCR treatment and plant biomass, standard error for those predictors, and p-values for those predictors. Negative predictor values indicate that the presence/increase of that predictor was associated with fewer EPN. Shaded areas indicate the 95% confidence interval.

## DISCUSSION

Infestation of roots by belowground herbivores can affect plant chemistry as plants utilize different strategies to combat these pests. Plants can alter their chemistry to reduce nutritional quality, produce toxic chemicals, and attract natural enemies in order to protect themselves from belowground pests (Turlings et al. 1995; Duffey and Stout 1996). Soil properties, soil communities, and plant genotypes can further complicate the plants’ chemical reactions in response to pests (Coley et al. 1985; Gouinguené and Turlings 2002; Degen et al. 2004). Here we show that belowground volatile production is significantly reduced in the presence of WCR, and maize genotype and cover crop legacy can interact to change belowground volatile profiles, both before and after infestation by western corn rootworm.

Volatile content produced during herbivore infestation was significantly lower compared to control volatile content (Fig. 2). This trend was true across almost every chemical class and each individual volatile that was identified (Figs. 4 and 5). Previous work looking at volatile production in aboveground systems has generally found the opposite to be true, namely that volatile production is increased in response to herbivore feeding (Dicke and Baldwin 2010). These changes have often been associated with the attraction of natural enemies which control the populations of these pests or the repulsion of conspecific herbivores (Rowen and Kaplan 2016).

While counterintuitive, a reduction in overall volatile production in response to root herbivore feeding could still be used by the plant and other organisms as a signaling mechanism. When dealing with specialist herbivores, induced volatile production has been shown to be a liability due to the increased attraction of specialist feeders (Kalberer et al. 2001; Kigathi et al. 2013). Granted the long history of maize monocultures in the U.S., it is possible that this reduction in root volatiles in response to herbivory is an adaptive response to reduce attraction of additional specialist root herbivores and therefore reduce feeding damage. A reduction in volatile emission in response to herbivory may also be energetically conservative especially when planted in monocultures with genetically similar plants. In a collective, contributing signals from individual plants are more efficient when each individual is contributing a lower quantity (Kigathi et al. 2013). Similar to results presented here, Kigathi et al. (2013) found that planting conspecifics together reduced total and herbivore-mediated aboveground volatile emissions as compared to one plant growing alone or planting two competing species together. It is also crucial to consider that specialist herbivores might manipulate the plant’s volatile production for their own benefit. By reducing the emission of volatiles, these herbivores can stay hidden from predators or parasitoids that rely on plant signals to locate their prey. This reduced volatile production can help them evade detection, effectively providing a “cloaking” mechanism. Moreover, by limiting volatiles, specialist herbivores may reduce intraspecific competition, as fewer competitors are attracted to the plant. This dual benefit of hiding from predators and reducing competition could explain why specialist herbivores might actively suppress volatile production in their host plants.

Alternatively, root damage by WCR could have led to volatile suppression via tissue loss, though this would contrast with most above and belowground studies which found that tissue loss leads to increased volatile emissions (Rasmann et al. 2005; Wenke et al. 2010; Skoczek et al. 2017). Soil properties could also be altered by WCR presence, masking volatile emissions. For example, the presence of WCR could signal to the plant to alter the flow of nutrients and water from plant to soil, changing the physical properties of the soil which could lead to volatile masking. We did not test soil physical properties before and after the addition of WCR due to the nature of our collection methods, but future studies could investigate this soil masking hypothesis.

WCR-mediated volatile production was also significantly affected by the combination of cover crop legacy and maize variety. After the radish cover crop treatment, 42C87 maize had a significant reduction in the majority of volatiles after WCR infestation, while MC4050 maize had almost no changes in volatile production (Fig. 5). Similarly, there were significant differences between constitutive volatiles and induced volatiles in MC4050 when planted after the cover crop mixture, but not in 42C87 (Fig. 5). These results indicate that each variety responded differently to the same initial conditions in the soil, and this also affected the induced volatile production after WCR-infestation. It is also possible that WCR may have fed differentially on the two varieties of maize depending on the cover crop, and reduced volatile production was a result of tissue loss.

Plants employ a variety of strategies to survive herbivore pressure, it is therefore possible that the two maize varieties in this study used different strategies in response to WCR feeding that required different environmental conditions. Given the proper nutrients, maize plants may employ a more confrontational strategy using secondary metabolites that require nutrients in scarce supply (Liang et al. 2013). On the other hand, should there be a nutrient deficit, an avoidant strategy may be more appropriate. Maize grown in the radish, triticale, and mixture soils all had significant changes in volatile production for both varieties, and nutrient analysis indicated that these soils also had the lowest percent organic matter (Trase et al. 2023). Maize grown after pea and fallow had almost no changes in volatile production in either variety and had relatively high percent organic matter (Trase et al. 2023). Generally, microbial biomass increases along with organic matter (Rice et al. 1997), suggesting that differences in soil microbial activity between cover crop regimes may be partially responsible for changes in herbivore-induced volatiles.

Multiple studies have explored the role of herbivore-induced plant volatiles in attracting natural enemies to the plant (Hare 2011). Here, we show that the entomopathogenic nematode, *S. feltiae*, does not overwhelmingly move toward maize plants infested with neonate western corn rootworm larvae despite the differences in volatiles between WCR-infested and control plants. Within this system, Rasmann et al. (2005), have identified (E)-β-caryophyllene as a compound that is produced by maize roots in response to WCR feeding and that this compound is attractive to multiple entomopathogenic nematodes which are natural enemies of WCR larvae. Importantly, Rasmann et al. (2005) also found that the production of this compound was lost in commercial U.S. varieties but was still present in European varieties. However, we detected (*E*)-β-caryophyllene in all of our treatments, and levels were significantly higher in control treatments in comparison to WCR-infested treatments (Fig. S1; *X*^2^_(1, *N* =375)_ = 38.35, *p* < 0.001). Rather than a lack of (*E*)-β-caryophyllene, there seems to be a decoupling of this volatile and EPN preference given that it was found in much higher levels in control plants and EPN did not preferentially move toward these plants. Another reason we might not have found similar volatile induction was the volatile collection method; instead of washing and grinding the roots in liquid nitrogen and collecting volatiles using SPME as seen in the Rasmann et al. (2005), volatiles were collected from live roots and soil in HaySep-Q adsorbent VOC traps.

The lack of significant upregulation in the volatile classes or individual volatile identified in this study in response to WCR feeding suggests that these hybrids may lack the genetic machinery necessary to attract entomopathogenic nematodes. Otherwise, WCR-related effectors might be affecting plant secondary metabolite production to reduce toxicity or apparency toward other herbivores, predators and other natural enemies. Previous work has explored how herbivore feeding in multiple systems can suppress plant defenses through oral secretions, frass, or associated endosymbionts (reviewed in Acevedo et al. 2015), though there is conflicting evidence for this type of suppression in the maize-WCR interaction (Barr et al. 2010; Robert et al. 2013). In terms of volatile emission, Peñaflor et al. (2011) found that oviposition by fall armyworm moths suppressed aboveground volatile emissions following larval herbivore in maize, lending support to the theory that certain herbivore-associated molecules may act to suppress volatile emissions.

Importantly, we did find that the lack of menthol was a strong predictor of WCR presence according to a random forest classification algorithm. While neither menthol nor its associated essential oils have been studied in depth in relation to the maize-WCR interaction, previous work with other coleopterans have found that menthol and/or peppermint extract were toxic to various weevils and common pollen beetles (reviewed in Kalemba and Synowiec 2019), suggesting menthol may have some insecticidal effects on WCR as well. Although maize itself is not known to produce menthol, its presence in the soil surrounding maize roots could be explained by microbial activity. Soil- or root-associated microbes may convert larger plant-derived terpenoids into menthol through enzymatic transformations. Several microbial taxa, including *actinomycetes*, *Rhodococcus*, and engineered strains of *E. coli* or *Saccharomyces cerevisiae*, have been shown to bioconvert monoterpenes such as limonene or menthone into menthol and menthol-like compounds (van der Werf et al. 1999; Duetz et al. 2003; Toogood et al. 2015; Lv et al. 2022; Maltseva et al. 2024; Ma et al. 2024). These microbes may utilize maize-released terpenoids or organic exudates as substrates, especially in the rhizosphere where such compounds accumulate. Notably, we observed that both menthol and other volatile organic compounds decrease in the presence of WCR herbivory, suggesting that root damage may suppress microbial biosynthesis, alter root exudation patterns, or disrupt microbial community composition. This highlights a potential multitrophic interaction between plants, herbivores, and microbes that shape belowground volatile profiles.

It is possible that EPN foraging strategy could differentially affect chemotactic success. Having an intermediate foraging strategy means that *S. feltiae* likely invest fewer resources into olfactory detection of insects that are further away. In a similar study using a larger six-choice olfactometer, Rasmann and Turlings (2008) found that not a single infective juvenile of *S. feltiae* moved from the central chamber toward any of the arms which contained either plants alone or plants infested with WCR (Rasmann and Turlings 2008). They hypothesized that this nematode, due to using an intermediate foraging strategy as opposed to a cruising-only strategy, does not use long-range chemical cues to find hosts but rather relies on shorter-range cues (Rasmann and Turlings 2008). While the nematodes in our study did move at least a few centimeters out of the central area of the olfactometer, the infective juveniles moved equally toward infested and non-infested plants suggesting they were not responding to obvious chemotactic signals.

Even at shorter ranges, entomopathogenic nematodes require insect-specific cues to locate their hosts. One explanation for the lack of chemotactic recognition by *S. feltiae* in this study could be that the neonate WCR were too small to emit a detectable signal. Kurtz et al. (2008) found that *S. feltiae* were able to successfully locate and kill ~ 60% of first instar WCR larvae, ~ 40% of second instars, and ~ 70% of third instars, suggesting that size and larval instar are not strong predictors of *S. feltiae* host-finding capabilities (Kurtz et al. 2008).

This is the first study to report a reduction in belowground volatile production in response to WCR feeding, especially in the monoterpenes and monoterpenoids. In addition, we show that previously planted cover crop treatment in combination with maize genotype can intensify this response, highlighting the importance of including genotype × environment interactions while studying plant-herbivore interactions, especially belowground. The lack of significant movement toward their hosts does not necessarily indicate that *S. feltiae* would be ineffective as a control for WCR, as we observed that overall they did disperse toward infested plants. Understanding when and how different entomopathogenic nematodes find their hosts will help inform how these nematodes are commercialized and used in agriculture.

## Supplementary Information

Below is the link to the electronic supplementary material.

ESM 1 (DOCX 135 KB)

ESM 2 (DOCX 16.4 KB)

## Data Availability

Data that support the findings of this study have been deposited in the following github repository: https://github.com/oliviatrase/Trase_etal_2025_Volatiles.
