# Supplementary material for: Suppression of Belowground Volatiles in Maize Depends on Cover Crop Legacy and Genotype
Source: J Chem Ecol. 2025 Aug 27;51(5):85. doi: 10.1007/s10886-025-01632-z (PMC12391165; doi:10.1007/s10886-025-01632-z)
Supplement: Supplementary file 1 — (DOCX 135 KB) [file 10886_2025_1632_MOESM1_ESM.docx]

Appendix S1

Suppression of belowground volatiles in maize depends on cover crop legacy and genotype

Journal of Chemical Ecology

Olivia W. Trase^1,2^, Nathaniel Mccartney^1^, Jared G. Ali^1,2^*

^1^ Department of Entomology, The Pennsylvania State University, University Park, PA, 16802, USA

^2^ Interdepartmental Degree Program in Ecology, The Pennsylvania State University, University Park, PA, 16802, USA

*Correspondence: [jga8@psu.edu](mailto:jga8@psu.edu)


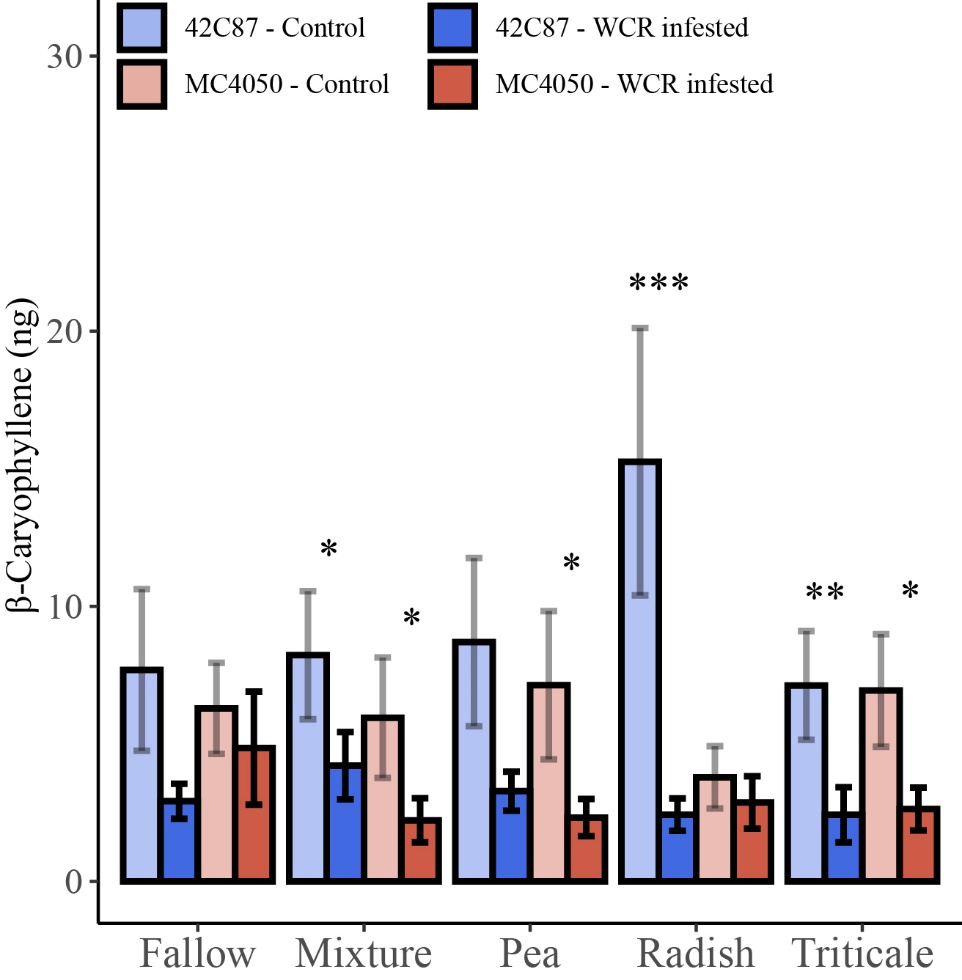


**Fig. S1 Bar plot displaying average quantities of β-Caryophyllene in each treatment.**

Volatile content was normalized by the internal standard (acetic acid nonyl ester) and is here expressed in terms of nanograms of compound. Bars represent ±1 SEM. Stars indicate where the difference between control and WCR infested treatments was significant (p < 0.05*, p < 0.01**, p < 0.001 ***).
