# Supplementary material for: Suppression of Belowground Volatiles in Maize Depends on Cover Crop Legacy and Genotype
Source: J Chem Ecol. 2025 Aug 27;51(5):85. doi: 10.1007/s10886-025-01632-z (PMC12391165; doi:10.1007/s10886-025-01632-z)
Supplement: Supplementary file 2 — (DOCX 16.4 KB) [file 10886_2025_1632_MOESM2_ESM.docx]

Appendix S2

Suppression of belowground volatiles in maize depends on cover crop legacy and genotype

Journal of Chemical Ecology

Olivia W. Trase^1,2^, Nathaniel Mccartney^1^, Jared G. Ali^1,2^*

^1^ Department of Entomology, The Pennsylvania State University, University Park, PA, 16802, USA

^2^ Interdepartmental Degree Program in Ecology, The Pennsylvania State University, University Park, PA, 16802, USA

*Correspondence: [jga8@psu.edu](mailto:jga8@psu.edu)

*Statistical Package Information*

Differences in total VOC content based on maize variety, cover crop treatment, and WCR infestation status were assessed using a linear mixed effects model (‘lme’ function from the ‘nlme’ package in R) followed by an ANOVA (‘anova’ function from the ‘stats’ package in R) where variety, cover crop treatment, and WCR status were fixed effects, and experimental block was a random effect.

Differences in total VOC content between infested and non-infested plants of each treatment were assessed using a Student’s T-test (‘lme’ function from the ‘nlme’ package followed by the ‘emmeans’ function from the ‘emmeans’ package in R).

Differences in volatile profiles were assessed using PERMANOVA (‘adonis2’ function from the ‘vegan’ package in R) and PERMDISP (‘vegdist’ function followed by the ‘betadisper’ function from the ‘vegan’ package in R). Experimental blocks were incorporating using the ‘setBlocks’ function in the ‘permute’ package in R.

For both individual compounds and chemical classes, differences between variety, cover crop treatment, and WCR infestation status were assessed using a generalized linear mixed model using a negative binomial distribution (‘glmmTMB’ function from the ‘glmmTMB’ package in R) followed by a type II ANOVA (‘Anova’ function from the ‘car’ package in R).

Prediction of WCR-feeding based on volatile composition was assessed using a random forest classification algorithm (‘RandomForestClassifier’ function from the ‘scikit-learn’ library and ‘ensemble’ module in Python).

Data was split into training data (80%) and testing data (20%) (‘train_test_split’ function from the ‘scikit-learn’ package and ‘model_selection’ module in Python).

The threshold for variance inflation factors (‘variance_inflation_factor’ function from the ‘stats_models’ package and ‘outliers_influence’ module in Python) was set to 10.

Best parameters for the random forest algorithm were selected using a grid search and ten iterations of cross-validation (‘GridSearchCV’ function from the ‘scikit-learn’ package and ‘model_selection’ module in Python).

Differences between total number of EPN recovered were assessed using ANOVA (‘lme’ function from the ‘nlme’ package followed by the ‘Anova’ function from the ‘car’ package in R).

Differences in average chemotaxis index between cover crop treatments were assessed using ANOVA (‘lme’ function from the ‘nlme’ package followed by the ‘Anova’ function from the ‘car’ package in R).

Differences in the number of EPN moving toward infested versus control plants for each cover crop treatment were assessed using a generalized linear mixed effects model with a Poisson distribution (‘glmer’ function from the ‘lme4’ package in R).
